# Rescue *in vitro* maturation may increase the pregnancy outcomes among women undergoing intracytoplasmic sperm injection

**DOI:** 10.3389/fendo.2022.1047571

**Published:** 2022-12-12

**Authors:** Dan-Yu Qin, Hua-Hua Jiang, Qing-Yun Yao, Wen Yao, Xiao-Qiong Yuan, Yi Wang, Tao-Ran Deng, Yao-Yao Du, Xin-Ling Ren, Na Guo, Yu-Feng Li

**Affiliations:** ^1^ Reproductive Medicine Center, Tongji Hospital, Tongji Medicine College, Huazhong University of Science and Technology, Wuhan, China; ^2^ Center for Reproductive Medicine, Department of Obstetrics and Gynecology, Peking University Third Hospital, Beijing, China

**Keywords:** rescue *in vitro* maturation, intracytoplasmic sperm injection, reproductive outcomes, live birth rate, cumulative live birth rate, propensity score matching

## Abstract

**Introduction:**

To investigate whether rescue in vitro maturation (R-IVM) improves the reproductive outcomes among women undergoing intracytoplasmic sperm injection (ICSI) after one oocyte retrieved cycle.

**Methods:**

Between January 2019 and December 2020, 2602 women who underwent ICSI in the Reproductive Medicine Center of Tongji Hospital, Wuhan, China, were included in our retrospective cohort study. There were 2112 women undergoing only ICSI and 490 women with R-IVM followed by ICSI. The intermediate reproductive outcomes and pregnancy outcomes were assessed, including the number of normally fertilized embryos, number of cleaved embryos, number of good-quality embryos, number of day-3 available embryos, number of embryos cultured past day-3, number of blastocysts, number of available blastocysts, biochemical pregnancy, miscarriage, clinical pregnancy and live birth. The perinatal outcomes were also assessed, including preterm birth and birth weight. The abovementioned outcomes were also calculated for in vivo matured and R-IVM oocytes separately in women undergoing ICSI with R-IVM group.

**Result(s):**

Compared with the women who underwent only ICSI, those who underwent ICSI with R-IVM had higher numbers of MII oocytes, normally fertilized embryos, cleaved embryos, day-3 available embryos, embryos cultured past day-3, and higher oocyte maturation rate, available embryo rate than women undergoing only ICSI. Additionally, we found that women undergoing ICSI with R-IVM had an increased chance of clinical pregnancy (adjusted OR=1.50, 95% CI: 1.17–1.93) and cumulative live birth (adjusted OR=1.35, 95% CI: 1.07–1.71). After propensity score matching (PSM), the cumulative live birth rate was 60.1% for women undergoing ICSI with R-IVM versus 54.9% for women undergoing only ICSI (OR=1.24, 95% CI: 0.94–1.63). The reproductive outcomes were also significantly different when calculated for in vivo matured and R-IVM oocytes separately in women undergoing ICSI with R-IVM group. All live births from R-IVM embryos were healthy and without malformations or complications.

**Conclusion:**

R-IVM may improve the reproductive outcomes of women undergoing ICSI. It may also provide a reference for the safety of R-IVM. This study maybe support a routine application of R-IVM among patients who intend to undergo ICSI.

## Introduction

Assisted reproductive technology (ART) is an effective and the final treatment for infertile couples. According to International Committee for Monitoring Assisted Reproductive Technologies (ICMART) annual world report, in 2014, the number of ART cycles reached 2.92 million globally and 1.15 million in China, 62.3% used intracytoplasmic sperm injection (ICSI) as the fertilization method (1, 2). An important aspect of the ICSI procedure was a precise determination of nuclear maturation status and oocyte morphology. About 15% of retrieved oocytes after controlled ovarian hyperstimulation (COH) are still immature and usually discarded (3). Women with a high proportion of immature oocytes after COH have a reduced chance of pregnancy or even cancel the cycle (4). Therefore, it is important to find ways to improve the utilization of immature oocytes.

*In vitro* maturation (IVM) is an alternative ART in which immature oocytes are retrieved at germinal vesicle(GV)/metaphase I(MI) stage and then matured *in vitro* to reach the metaphase II (MII) stage (5, 6). Generally, the classical IVM involves the immature oocytes from unstimulated or minimally stimulated cycles and applies to patients with polycystic ovary syndrome (PCOS) or patients with premature ovarian failure (POF) (7–9). According to the American Society for Reproductive Medicine(ASRM) committee opinion on IVM, a type of IVM known as rescue IVM (R-IVM) has been developed to benefit more infertile couples, which is the *in vitro* maturation of immature oocytes from conventional COH (10). Additionally, the zona pellucida of immature oocytes may harden and be completely exposed during IVM, so ICSI has been advocated as the preferred fertilization method for IVM oocytes (11).

Previous studies suggested that IVM is safe for infertile women, and for children born from the technique (12, 13). However, studies generally suggest that the development capacity of embryos derived from IVM oocytes is not comparable to sibling embryos derived from *in vivo* matured oocytes, which can still provide extra blastocysts for transfer (14–16). In addition, a previous study implied that women transferred with embryos from R-IVM oocytes can achieve live births, especially for those with poor prognoses (17). Some studies found that women with a high antral follicle count (AFC) after IVM treatment could achieve acceptable live birth rates and have no increased risk of adverse perinatal outcomes (18–20). There is no consistent opinion about the clinical application of R-IVM. It is still unclear whether ICSI with R-IVM increases reproductive outcomes after one oocyte retrieved cycle and supports routine utilization among all patients. Thus, we compared the reproductive outcomes of women undergoing conventional ICSI and ICSI with R-IVM to determine whether women undergoing ICSI with R-IVM increase reproductive outcomes after one oocyte retrieved cycle.

## Materials and methods

### Patient cohort

We performed a retrospective cohort study at the Reproductive Medicine Center of Tongji Hospital, Wuhan, China. Women aged 20-45 years who underwent ICSI cycles (with at least one mature oocyte and one immature oocyte) between January 2019 and December 2020 were included, and only one oocyte retrieved cycle per woman. Women chose between only ICSI or combined with R-IVM after consulting the ART specialist about the benefits and risks of each strategy. We excluded women with chromosomal abnormality (n=5), who underwent oocyte cryopreservation-thaw cycles (n=16), oocyte donation cycles (n=3), or preimplantation genetic testing (PGT) cycles (n=63). Finally, a total of 2602 women were included and divided into two groups according to the treatment strategy adopted (i.e., only ICSI or ICSI with R-IVM), with follow-up until July 2022. This study was approved by the ethics committee of Tongji Hospital, Tongji Medical College, Huazhong University of Science and Technology.

### ICSI procedures and *in vitro* maturation

Details on COH and ICSI procedures were described in our previous publications (21, 22). Briefly, COH protocols included gonadotropin-releasing hormone (GnRH) agonist, GnRH antagonist, luteal phase stimulation, and mild-stimulation protocols. During COH, we monitored serum hormone levels, follicle size, follicle count, and endometrial thickness. When at least two leading follicles reached ≥18 mm in diameter, recombinant HCG (Ovidrel; Merck-Serono) was then applied to trigger ovulation. The cumulus-oocyte complexes (COCs) were retrieved 36-38 hours after the HCG trigger. For the only ICSI group, cumulus cells of oocytes were removed 2 hours after retrieval with 80 IU hyaluronidase(Vitrolife, Sweden). And then nuclear maturation status of all denuded oocytes was assessed under an inverted microscope and classified as GV, MI, and MII stage. Only those oocytes at the MII stage were selected for ICSI while GV and MI oocytes were typically discarded. However, for the ICSI with R-IVM group, in addition to MII oocytes undergoing ICSI, those denuded immature oocytes, including GV and MI oocytes retrieved from COH cycles, were also used for further culture and utilization *in vitro*. They were subjected to rescue IVM treatment, which was continued to individually culture in G1-plus medium (Vitrolife, Sweden) at 37°C in a humidified atmosphere of 5% O2, 6% CO2, and 89% nitrogen for 24 hours. Moreover, it was necessary to monitor the maturity of immature oocytes periodically at 6 hours intervals until 24 hours. When immature oocytes that completed nuclear maturation during the preincubation period were selected for subsequent fertilization with the partner’s semen by ICSI.

Fresh embryo transfer (ET) was performed on day 3 after oocyte retrieval. The surplus embryos that were considered good or fair quality would be cryopreserved or extensively cultured to day 5 or 6 to reach the blastocyst stage for later transfer in subsequent frozen ET cycles. All the additional embryos were cryopreserved by vitrification using the Cryotop system. For a maximum of two day 3 embryos or one or two day 5-6 blastocysts were transferred according to the Code of Practice for Assisted Reproductive Technology developed by the Ministry of Health of the People’s Republic of China. Embryo transfers were performed by specified gynecologists with the same standard ET protocol.

### Study variables

Data on maternal age, body mass index (BMI), previous conventional IVF attempts, duration of infertility (years), cause of infertility (ovulation disorder, diminished ovarian reserve, tubal or pelvic factor, endometriosis, male factor, uterine factor, unexplained), type of infertility (primary or secondary), baseline follicle-stimulating hormone (FSH), AFC, anti-Müllerian hormone (AMH), stimulation protocol (agonist, antagonist or others), gonadotropin duration (days), gonadotropin dosage, E2 and P level on the day of hCG injection, endometrial thickness, number of oocytes retrieved, number of embryos transferred, stage of embryo transferred (cleavage embryo or blastocyst), and type of first transfer (fresh or frozen ET) were extracted from the medical records. BMI (kg/m^2^) was calculated as the weight in kilograms divided by the square of the height in meters. Ovulation disorder included PCOS and abnormal uterine bleeding (AUB). Male factors included semen abnormalities, coital infertility (i.e. erectile dysfunction and ejaculatory dysfunction). Infertility without a clear known cause was classified as unexplained infertility.

### Outcome assessments

The intermediate reproductive and pregnancy outcomes of ICSI treatment, including the number of normally fertilized embryos, number of cleaved embryos, number of good-quality embryos, number of day-3 available embryos, number of embryos cultured past day-3, number of blastocysts, number of available blastocysts, biochemical pregnancy, miscarriage, clinical pregnancy and live birth were abstracted from the medical records. The perinatal outcomes, including delivery mode (natural labor or cesarean delivery), fetal sex (male or female), gestational age, and birth weight were obtained from telephone interviews after delivery. The abovementioned outcomes were also calculated for *in vivo* matured and R-IVM oocytes separately in women undergoing ICSI with R-IVM group.

Normal fertilization was defined as the presence of two pronuclei (2PN). The normal fertilization rate was defined as the number of 2PN oocytes divided by the number of MII oocytes. The cleavage rate was defined as the number of cleaved embryos developed from 2PN oocytes divided by the number of 2PN oocytes. Cleavage embryonic development was evaluated using Veeck systems. Good-quality embryos were defined as normally fertilized embryos with 7-9 cells, fragmentation less than 10%, and without multinucleation on day-3. The good-quality embryo rate was defined as the number of good-quality embryos divided by the number of cleaved embryos. The available embryo rate was defined as the number of day-3 available embryos divided by the number of oocytes retrieved. The blastocysts were evaluated using the Gardner system. On Day 5 or 6, blastocysts with ≥3BC grade were considered to be available for cryopreservation. The blastocyst formation rate was defined as the blastocysts divided by the number of embryos cultured past day-3. The available blastocyst rate was defined as the number of blastocysts for cryopreservation divided by the number of embryos cultured past day-3. Biochemical pregnancy was defined as a positive result of HCG measurement without ultrasonographic visualization of clinical pregnancy. Clinical pregnancy was defined as the presence of a gestational sac with fetal heart activity by ultrasound 28 days after embryo transfer. Miscarriage was defined as pregnancy loss before gestational week 28. Live birth (LB) was defined as the delivery of a live newborn after gestational week 28. Cumulative LBR was defined as the chance of having LB after fresh and frozen transfers of embryos derived from one ICSI cycle. Preterm birth was defined as delivery at <37 weeks of gestation. Low birth weight was defined as child birth weight <2500g. Macrosomia was defined as child birth weight >4000g.

### Statistical analysis

Differences between groups were evaluated using Student’s t-tests, Mann-Whitney U test, Wilcoxon signed-rank test, chi-squared tests, or Fisher’s exact tests based on variable distribution and property. Results were reported as the mean ± SD or n (%).

Propensity score matching (PSM) was adapted to avoid confounding bias and excessive numerical difference. Confounders related to the reproductive outcomes were chosen based on literature review, including age, BMI, AFC, ovarian stimulation protocol, cause of infertility, previous conventional IVF attempts, number of oocytes retrieved, and oocyte maturation rate at retrieval. The propensity scores (PS) were estimated from a logistic regression model that considered the aforementioned confounders. Each woman who underwent only ICSI was matched (a 1:1 match) to a corresponding woman who underwent R-IVM followed by ICSI using an optimal matching algorithm by randomly selecting each pair with the closest PS. The caliper was set to 0.02.

Univariate generalized linear models were applied for the matched cohorts to evaluate the associations between treatment and reproductive outcomes. Multivariate generalized linear models were applied for the unmatched cohorts, and regression coefficients and 95% confidence intervals (CIs) adjusting for confounders were calculated. For intermediate reproductive outcomes, the obtained regression coefficients were converted into percentage changes using the following formula: [exp(β) -1] ×100%. For pregnancy outcomes, odds ratio (OR) and 95% CI were reported. The Kaplan-Meier method was used to estimate the cumulative LBR, and comparisons of cumulative LBR were made using the log-rank test. We performed a subgroup analysis based on women with or without diminished ovarian reserve (DOR). We also investigated the modification effects by type of infertility (primary and secondary). To test the robustness of our results, we conducted several sensitivity analyses. First, we reanalyzed the data by excluding women with PCOS. Second, we reanalyzed the data after excluding cycles from couples diagnosed with male infertility only. Statistical analysis was conducted using SPSS 22.0 software and R 4.2.1 software. Statistical significance was defined as a *P*-value <0.05.

## Results

### Demographics

**Table 1** shows the demographic characteristics. Between January 2019 and December 2020, 2602 women undergoing ICSI in our center were included in this analysis (**Figure S1**). There were 2112 women undergoing only ICSI and 490 women with R-IVM followed by ICSI. There were significant differences between only ICSI and ICSI with R-IVM in terms of BMI (21.9 ± 3.1 versus 22.4 ± 3.2, *P*<0.01), previous conventional IVF attempts, stimulation protocol, gonadotrophin duration, number of retrieved oocytes, and oocyte maturation rate at retrieval. After PSM, 838 women (419 women in each group) were included in the analysis, and none of the demographic characteristics demonstrated a significant difference between groups. And distributions of PS and standard differences indicated a balance between the compared cohorts (**Figure S2**).

**Table 1:** Patient characteristics before and after propensity score matching.

Characteristic	Before PSM			After PSM		
	Only ICSI	ICSI withR-IVM	*P*	Only ICSI	ICSI withR-IVM	*P*
Number	2112	490		419	419	
Age (years)	30.9 ± 4.3	31.2 ± 4.5	0.09	31.0 ± 4.7	31.2 ± 4.6	0.57
BMI	21.9 ± 3.1	22.4 ± 3.2	0.00*	22.2 ± 3.1	22.2 ± 3.0	0.77
Duration of infertility	3.4 ± 2.5	3.5 ± 2.6	0.29	3.4 ± 2.8	3.5 ± 2.5	0.75
Previous conventional IVF attempts						
1	1483 (70.2)	310 (63.3)		283 (67.5)	284 (67.8)	
2	442 (20.9)	127 (25.9)		98 (23.4)	94 (22.4)	
≥3	187 (8.9)	53 (10.8)	0.01*	38 (9.1)	41 (9.8)	0.91
Cause of infertility						
Ovulation disorder	158 (7.5)	51 (10.4)		38 (9.1)	41 (9.8)	
DOR	294 (13.9)	70(14.3)		63 (15.0)	58 (13.8)	
Tubal or pelvic factor	535 (25.3)	129 (26.3)		103 (24.6)	103 (24.6)	
Endometriosis	57 (2.7)	13 (2.7)		13 (3.1)	11 (2.6)	
Male factor	927 (43.9)	195 (39.8)		179 (42.7)	180 (43.0)	
Uterine factor	53 (2.5)	9 (1.8)		5 (1.2)	6 (1.4)	
Unexplained	88 (4.2)	23 (4.7)	0.32	18 (4.3)	20 (4.8)	0.99
Type of infertility						
Primary	1625 (76.9)	362 (73.9)		317 (75.7)	316 (75.4)	
Secondary	487 (23.1)	128 (26.1)	0.15	102 (24.3)	103 (24.6)	0.94
FSH (mIU/ml)	7.5 ± 2.5	7.5 ± 2.4	0.70	7.5 ± 2.6	7.4 ± 2.4	0.54
AFC	13.8 ± 7.0	13.9 ± 7.5	0.91	13.5 ± 6.9	14.1 ± 7.5	0.27
AMH (ng/ml)	4.8 ± 3.9	4.7 ± 3.9	0.61	4.6 ± 3.7	4.8 ± 3.9	0.53
Stimulation protocol						
Agonist	1159 (54.9)	225 (45.9)		207 (49.4)	207 (49.4)	
Antagonist	776 (36.7)	216 (44.1)		167 (39.9)	174 (41.5)	
Others	177 (8.4)	49 (10.0)	0.00*	45 (10.7)	38 (9.1)	0.69
Gn duration (days)	10.0 ± 1.8	9.7 ± 2.0	0.02*	9.8 ± 1.8	9.8 ± 2.0	0.80
Gn dosage (IU)	2414.5 ± 869.2	2412.6 ± 885.4	0.97	2463.0 ± 873.0	2367.6 ± 849.1	0.11
E_2_ on HCG day (pg/ml)	2761.8 ± 1694.9	2605.2 ± 1690.2	0.07	2557.8 ± 1575.4	2661.1 ± 1713.5	0.36
P on HCG day (ng/ml)	0.9 ± 0.9	0.8 ± 0.5	0.12	0.8 ± 0.7	0.8 ± 0.5	0.55
Endometrial thickness (mm)	11.5 ± 2.6	11.3 ± 2.8	0.21	11.4 ± 2.8	11.3 ± 2.8	0.61
No. of oocytes retrieved	14.1 ± 7.2	12.8 ± 6.7	0.00*	13.2 ± 7.0	13.3 ± 6.8	0.76
Oocyte maturation rate at retrieval (%)	75.1 ± 13.7	61.8 ± 19.7	0.00*	66.1 ± 16.3	66.6 ± 16.6	0.66

Data are presented as mean ± standard deviation or N (%). BMI = body mass index; DOR = diminished ovarian reserve; FSH = follicle-stimulating hormone; AFC = antral follicle count; AMH = anti-Müllerian hormone; Gn = gonadotropin; HCG = human chorionic gonadotrophin; PSM = propensity score matching; R-IVM = rescue in vitro maturation; ICSI = intracytoplasmic sperm injection.

*Statistically significant, with P < 0.05.

### Intermediate reproductive outcomes

The intermediate reproductive outcomes are shown in **Table 2** and **Table S1**. Before matching, compared with the women who underwent only ICSI, the women who underwent ICSI with R-IVM had higher numbers of MII oocytes (16.7%; 95%CI: 12.9% to 20.5%), normally fertilized embryos (14.3%; 95%CI: 9.8% to 18.9%), cleaved embryos (12.7%; 95%CI: 8.2% to 17.3%), good-quality embryos (6.7%; 95%CI: 0.4% to 13.3%), day-3 available embryos (12.8%; 95%CI: 8.3% to 17.5%), embryos cultured past day-3 (16.2%; 95%CI:11.0% to 21.6%), blastocysts (12.5%; 95%CI:6.2% to 19.1%), available blastocysts (9.2%; 95%CI:1.9% to 16.9%), oocyte maturation rate (113.9%; 95%CI: 98.2% to 131.0%), available embryo rate (27.8%; 95%CI: 20.4% to 35.6%), after adjustment for confounders. The good-quality embryo rate was lower (–9.5%; 95%CI: –16.6% to –1.9%) among women undergoing ICSI with R-IVM. After PSM, compared with the women who underwent only ICSI, the women who underwent ICSI with R-IVM had higher numbers of MII oocytes (18.2%; 95%CI: 13.2% to 23.5%), normally fertilized embryos (15.5%; 95%CI: 9.6% to 21.8%), cleaved embryos (9.9%; 95%CI: 4.2% to 15.9%), day-3 available embryos (14.8%; 95%CI: 8.7% to 21.2%), embryos cultured past day-3 (15.2%; 95%CI:8.4% to 22.5%), oocyte maturation rate (82.8%; 95%CI: 67.7% to 99.2%), available embryo rate (27.8%; 95%CI: 18.6% to 37.7%).

**Table 2:** Intermediate reproductive outcomes of women undergoing only ICSI versus women undergoing ICSI with R-IVM.

Outcome	Before PSM		After PSM	
	Percent change (95%CI)^a^	*P*	Percent change (95%CI)	*P*
MII oocytes	16.7 (12.9, 20.5)	0.00*	18.2 (13.2, 23.5)	0.00*
Oocyte maturation rate (%)	113.9 (98.2, 131.0)	0.00*	82.8 (67.7, 99.2)	0.00*
Normally fertilized embryos	14.3 (9.8, 18.9)	0.00*	15.5 (9.6, 21.8)	0.00*
Normal fertilization rate (%)	–4.0 (–10.5, 3.0)	0.26	–5.6 (–13.9, 3.6)	0.23
Cleaved embryos	12.7 (8.2, 17.3)	0.00*	9.9 (4.2, 15.9)	0.00*
Cleavage rate (%)	–4.7 (–25.6, 22.2)	0.71	–20.0 (–43.5, 13.3)	0.21
Good-quality embryos	6.7 (0.4, 13.3)	0.04*	5.0 (–3.0, 13.6)	0.23
Good-quality embryo rate (%)	–9.5 (–16.6, –1.9)	0.02*	–9.4 (–18.6, 0.9)	0.07
Day-3 available embryos	12.8 (8.3, 17.5)	0.00*	14.8 (8.7, 21.2)	0.00*
Available embryo rate (%)	27.8 (20.4, 35.6)	0.00*	27.8 (18.6, 37.7)	0.00*
Embryos cultured past day-3	16.2 (11.0, 21.6)	0.00*	15.2 (8.4, 22.5)	0.00*
Blastocysts	12.5 (6.2, 19.1)	0.00*	3.6 (–3.9, 11.8)	0.36
Blastocyst formation rate (%)	4.4 (–5.1, 15.0)	0.38	–5.6 (–17.0, 7.3)	0.38
Available blastocysts	9.2 (1.9, 16.9)	0.01*	2.4 (–6.5, 12.2)	0.61
Available blastocyst rate (%)	–3.1 (–11.6, 6.4)	0.51	–5.7 (–16.6, 6.7)	0.35

Data are presented as mean ± standard deviation. BMI = body mass index; AFC = antral follicle count; MII = metaphase 2; PSM = propensity score matching; ICSI = intracytoplasmic sperm injection; R-IVM = rescue in vitro maturation.

a Adjusted for age, BMI, AFC, ovarian stimulation protocol, cause of infertility, previous conventional IVF attempts, number of oocytes retrieved, and oocyte maturation rate at retrieval.

*Statistically significant, with P < 0.05.

The intermediate reproductive outcomes were also calculated for *in vivo* matured and R-IVM oocytes separately in women undergoing ICSI with R-IVM group (**Table S3**). Compared with *in vivo* matured oocytes, combined R-IVM and *in vivo* matured oocytes group had significant higher numbers of MII oocytes, normally fertilized embryos, cleaved embryos, good-quality embryos, day-3 available embryos, embryos cultured past day-3, blastocysts, available blastocysts, had lower normal fertilization rate.

### Pregnancy and perinatal outcome

The pregnancy outcomes are shown in **Table 3** and **Table S2**. Before matching, compared with the women who underwent only ICSI, those who underwent ICSI with R-IVM had an increased chance of clinical pregnancy after the fresh ET (adjusted OR=1.32, 95% CI: 1.00–1.73), after the first ET (adjusted OR=1.36, 95% CI: 1.09–1.70). The live birth rate after the first ET was 42.0% for women undergoing ICSI with R-IVM versus 42.3% for women undergoing only ICSI (adjusted OR=1.21, 95% CI: 0.97–1.51). Women undergoing ICSI with R-IVM had an increased chance of cumulative clinical pregnancy (adjusted OR=1.50, 95% CI: 1.17–1.93), and cumulative live birth (adjusted OR=1.35, 95% CI: 1.07–1.71).

**Table 3:** Pregnancy outcomes of women undergoing only ICSI versus women undergoing ICSI with R-IVM.

Outcome	Before PSM			After PSM		
	Only ICSI	ICSI with R-IVM	Adjusted OR (95% CI)	Only ICSI	ICSI withR-IVM	OR (95% CI)
**The first ET (n)**	2026	462		387	399	
No. of embryos transferred	1.2 ± 0.4	1.2 ± 0.4		1.2 ± 0.4	1.2 ± 0.4	
1	1647 (81.3)	375 (81.2)		308 (79.6)	324 (81.2)	
2	379 (18.7)	87 (18.8)		79 (20.4)	75 (18.8)	
Stage of embryos transferred						
Cleavage embryo	1401 (69.2)	351(76.0)		292 (75.5)	295 (73.9)	
Blastocyst	625 (30.8)	111(24.0)		95 (24.5)	104 (26.1)	
Type of first transfer						
Fresh ET	1286 (63.5)	288 (62.3)		253 (65.4)	250 (62.7)	
Frozen ET	740 (36.5)	174 (37.7)		134 (34.6)	149 (37.3)	
Biochemical pregnancy	51 (2.5)	16 (3.5)	1.55 (0.81, 2.96) ^a^	9 (2.3)	16 (4.0)	1.76 (0.77, 4.02)
Clinical pregnancy	1020 (50.3)	245 (53.0)	**1.36 (1.09, 1.70)** ^a^	167 (43.2)	215 (53.9)	**1.54 (1.16, 2.04)**
Miscarriage	164 (8.1)	51 (11.0)	1.40 (0.98, 2.00) ^a^	22 (5.7)	44 (11.0)	**2.06 (1.21, 3.50)**
Live birth	856 (42.3)	194 (42.0)	1.21 (0.97, 1.51) ^a^	145 (37.5)	171 (42.9)	1.25 (0.94, 1.67)
Singleton	816 (95.3)	187 (96.4)		140 (96.6)	167 (97.7)	
Twin	40 (4.7)	7 (3.6)		5 (3.4)	4 (2.3)	
**Cumulative outcome (n)**	2112	490		419	419	
No. of transfers	1.2 ± 0.5	1.2 ± 0.5		1.2 ± 0.5	1.2 ± 0.5	
1	2026	462		387	399	
2	756	145		150	136	
3	182	35		29	34	
≥4	54	7		9	7	
Biochemical pregnancy	103 (4.9)	26 (5.3)	1.54 (0.92, 2.60) ^b^	16 (3.8)	24 (5.7)	1.53 (0.80, 2.93)
Clinical pregnancy	1459 (69.1)	322 (65.7)	**1.50 (1.17, 1.93)** ^b^	256 (61.1)	290 (69.2)	**1.43 (1.08, 1.91)**
Miscarriage	173 (8.2)	44 (9.0)	1.18 (0.81, 1.73) ^b^	26 (6.2)	38 (9.1)	1.51 (0.90, 2.53)
Cumulative live birth	1286 (60.9)	278 (56.7)	**1.35 (1.07, 1.71)** ^b^	230 (54.9)	252 (60.1)	1.24 (0.94, 1.63)
Singleton	1200 (93.3)	264 (95.0)		214 (93.0)	241 (95.6)	
Twin	86 (6.7)	14 (5.0)		16 (7.0)	11 (4.4)	

Data are presented as mean ± standard deviation or N (%). PSM = propensity score matching; ICSI = intracytoplasmic sperm injection; R-IVM = rescue in vitro maturation, OR= odds ratio.

a Adjusted for age, BMI, ovarian stimulation protocol, previous conventional IVF attempts, number of oocytes retrieved, oocyte maturation rate at retrieval, and stage of embryos transferred.

b Adjusted for age, BMI, ovarian stimulation protocol, previous conventional IVF attempts, number of oocytes retrieved, oocyte maturation rate at retrieval, and number of transfers.

The bold values indicate statistically significant (P < 0.05).

**Figure 1:**
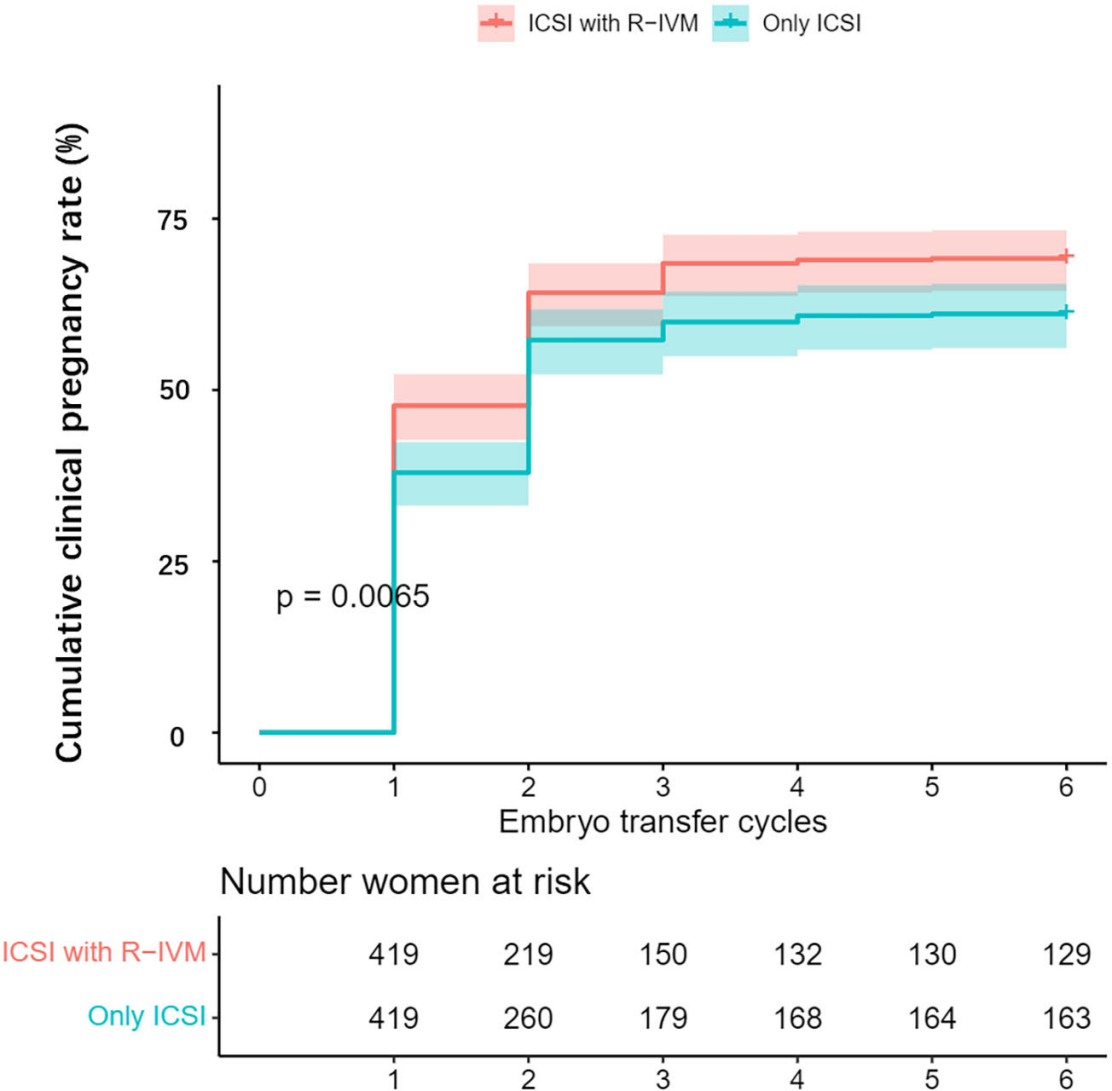
Kaplan-Meier curve for the cumulative clinical pregnancy rate after propensity score matching.

**Figure 2:**
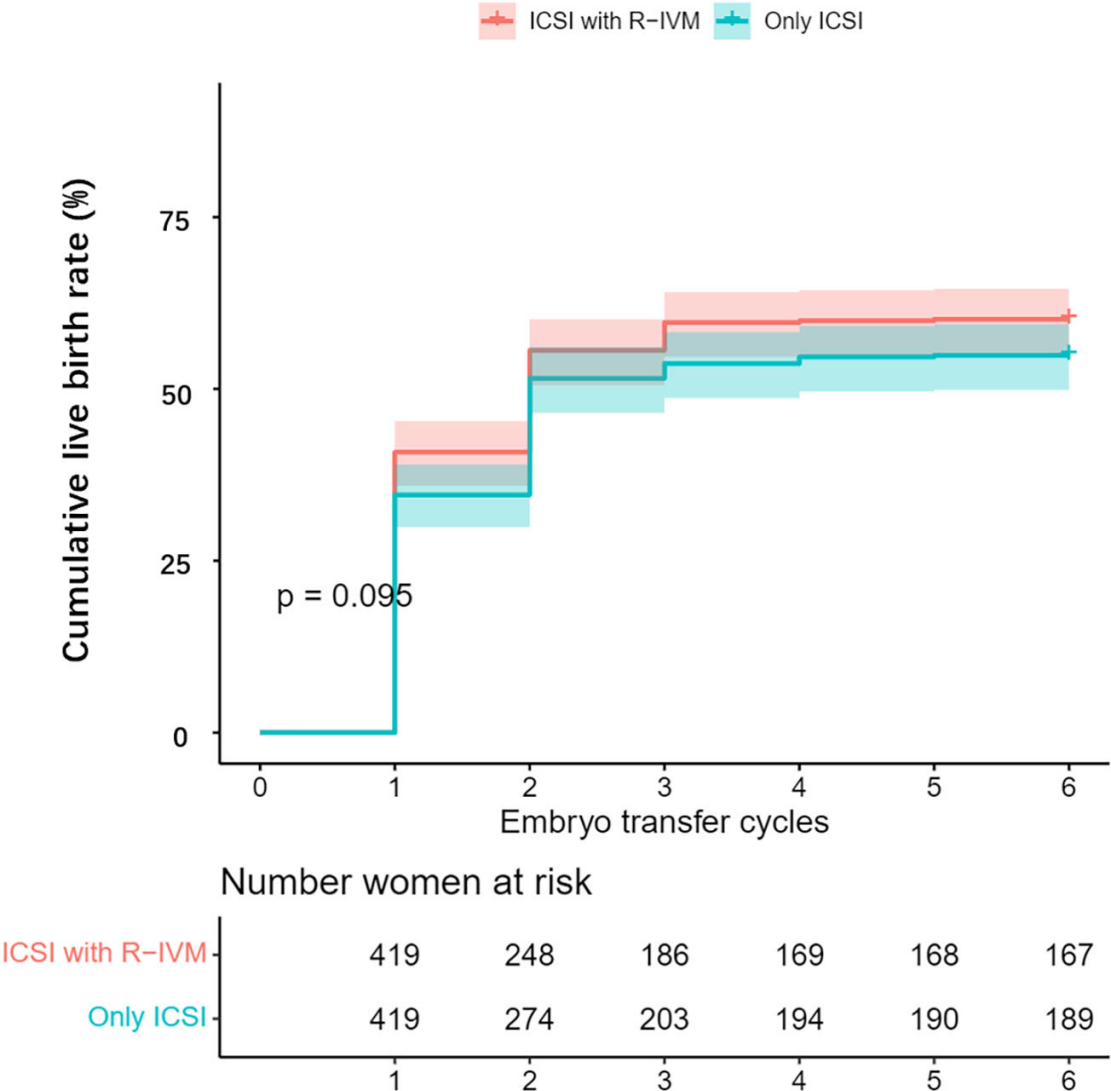
Kaplan-Meier curve for the cumulative live birth rate after propensity score matching.

After PSM, compared with the women who underwent only ICSI, those who underwent ICSI with R-IVM had an increased chance of clinical pregnancy after the fresh ET cycle (OR=1.43, 95% CI: 1.01–2.03), after the first ET (OR=1.54, 95% CI: 1.16–2.04), and miscarriage (OR=2.06, 95% CI: 1.21–3.50) after the first ET. The live birth rate after the first ET was 42.9% for women undergoing ICSI with R-IVM versus 37.5% for women undergoing only ICSI (OR=1.25, 95% CI: 0.94–1.67). Women undergoing ICSI with R-IVM had an increased chance of cumulative clinical pregnancy (OR=1.43, 95% CI: 1.08–1.91; **Figure 1**). The cumulative live birth rate was 60.1% for women undergoing ICSI with R-IVM versus 54.9% for women undergoing only ICSI (OR=1.24, 95% CI: 0.94–1.63; **Figure 2**).

The perinatal outcomes of all live birth in the two group are shown in **Table 4**. There were no significant differences between the two groups in terms of delivery mode, gestational age, birth weight, preterm birth rate, macrosomia, and low birth weight.

**Table 4:** Neonatal outcomes of women undergoing only ICSI and women undergoing ICSI with R-IVM.

Outcome	Before PSM		After PSM	
	Only ICSI	ICSI with R-IVM	Only ICSI	ICSI with R-IVM
Number	1286	278	1286	278
Delivery mode				
Natural labor	281 (21.9)	49 (17.6)	281 (21.9)	49 (17.6)
Cesarean delivery	1005 (78.1)	229 (82.4)	1005 (78.1)	229 (82.4)
Gender				
Male	692	144	692	144
Female	680	148	680	148
Gestational age (weeks)	38.1 ± 1.8	38.0 ± 2.0	38.1 ± 1.8	38.0 ± 2.0
Birth weight (g)	3239.0 ± 529.4	3199.8 ± 571.1	3239.0 ± 529.4	3199.8 ± 571.1
Preterm birth < 37 wk	169 (13.1)	45 (16.2)	169 (13.1)	45 (16.2)
Macrosomia > 4,000 g	73 (5.7)	15 (5.4)	73 (5.7)	15 (5.4)
Low birth weight < 2,500 g	103 (8.0)	26 (9.4)	103 (8.0)	26 (9.4)
**Singleton**				
Number	1200	264	1200	264
Delivery mode				
Natural labor	278 (23.2)	48 (18.2)	278 (23.2)	48 (18.2)
Cesarean delivery	922 (76.8)	216 (81.8)	922 (76.8)	216 (81.8)
Gender				
Male	612 (51.0)	132 (50.0)	612 (51.0)	132 (50.0)
Female	588 (49.0)	132 (50.0)	588 (49.0)	132 (50.0)
Gestational age (weeks)	38.3 ± 1.6	38.1 ± 1.9	38.3 ± 1.6	38.1 ± 1.9
Birth weight (g)	3297.6 ± 483.5	3244.9 ± 539.3	3297.6 ± 483.5	3244.9 ± 539.3
Preterm birth < 37 wk	109 (9.1)	34 (12.9)	109 (9.1)	34 (12.9)
Macrosomia > 4,000 g	73 (6.1)	15 (5.7)	73 (6.1)	15 (5.7)
Low birth weight < 2,500 g	51 (4.3)	16 (6.1)	51 (4.3)	16 (6.1)

Data are presented as mean ± standard deviation or N (%). ICSI = intracytoplasmic sperm injection; R-IVM = rescue in vitro maturation.

The pregnancy and perinatal outcome were also calculated for *in vivo* matured and R-IVM oocytes separately in women undergoing ICSI with R-IVM group (**Table S4**). Compared with *in vivo* matured oocytes, combined R-IVM and *in vivo* matured oocytes group had higher cumulative clinical pregnancy rate and cumulative live birth rate. Sixty-seven cases transferred embryos originated from R-IVM oocytes, 33 cases had pregnancy, 27 cases had live birth. In addition, only 17 cases transferred embryos originated from mixed *in vivo* matured and R-IVM oocytes, 9 cases had pregnancy, 8 cases had live birth, and one case had twin delivery. All live births from R-IVM embryos were healthy and without malformations or complications.

### Sensitivity analysis

The abovementioned results were largely unchanged when we restricted the analysis to the women without DOR (**Table S5**), to women with primary infertility (**Table S6**), to women without PCOS (**Table S7**), or when the cycles from the couples with male infertility only were excluded (**Table S8**). However, when we restricted our analysis to women with DOR, significant differences can be only observed in MII oocytes and oocyte maturation rate, and 2 live births were achieved from 7 cycles that transfer embryos from R-IVM; when we restricted our analysis to women with secondary infertility, significant differences can be only observed in oocyte maturation rate and available embryo rate.

## Discussion

In this study, we observed that women undergoing ICSI with R-IVM had higher numbers of MII oocytes, normally fertilized embryos, cleaved embryos, day-3 available embryos, embryos cultured past day-3, and had higher oocyte maturation rate, available embryo rate than women undergoing only ICSI. Additionally, we found that women undergoing ICSI with R-IVM had an increased chance of clinical pregnancy and cumulative live birth. The reproductive outcomes were also significantly different when calculated for *in vivo* matured and R-IVM oocytes separately in women undergoing ICSI with R-IVM group. All live births from R-IVM embryos were healthy and without malformations or complications.

Consistent with our results, previous studies also found that the fertilization rate and good-quality embryo rate were significantly lower in the R-IVM followed by ICSI group (23–25). These results suggest that post-IVM nuclear maturation is morphologically complete, but cytoplasmic maturation is insufficient or incomplete. Insufficient ooplasmic maturation inhibits the release of cortical granules into the perivitelline space, resulting in zona hardening with consequent interference with fertilization and blastocyst development (26–30). Additionally, the oocytes cultured in the medium for an extended period may lead to oocyte aging and poorer embryonic development quality (31–33). There were different results in reproductive outcomes between groups when we restricted the analyses to women with secondary infertility or with DOR, which also can be explained by their older age (secondary infertility: 33.6 ± 4.9 years, DOR: 34.6 ± 5.4 years). However, other studies reported that the numbers of cleaved embryos, good-quality embryos, day-3 available embryos, and the available embryo rate were similar between groups (34–36). This inconsistency with our results may be related to the differences in various IVM protocols, with oocyte aspiration performed in unstimulated cycles or stimulated cycles, and with or without an HCG trigger.

Our study showed that women undergoing ICSI with R-IVM had an increased chance of clinical pregnancy and cumulative live birth. Inconsistent with our results, a few observational studies found that cumulative rates of clinical pregnancy and live birth were significantly lower in the IVM group compared with the standard ICSI group (19, 20, 37). The inconsistency may be related to the differences in the study population. In addition, the proportion of MII oocytes can be affected by ovarian stimulation protocols (38). The IVM in our study involved the oocytes from conventional COH cycles, but the IVM in other studies involved the oocytes from unstimulated or minimally stimulated cycles. And we cultured immature oocytes in a standard G1-plus medium, but they cultured immature oocytes in a pre-maturation medium supplemented with recombinant FSH. Regarding the recently traditional IVM research, the application of a pre-maturation step, a biphasic IVM culturing system, and adjustments to cumulus cell removal or culture media/conditions have a marked effect on the clinical outcomes of IVM oocytes (39–42). The higher success rate of women undergoing ICSI with R-IVM in our study could be also explained by the higher numbers of cleaved embryos, good-quality embryos, and day-3 available embryos. The clinical pregnancy and live birth rate after the first ET were higher among women undergoing ICSI with R-IVM, which may be an additive effect of the higher cumulative live birth rate. The oocyte maturation rate at retrieval was associated with the live birth rate (4, 43). Accordingly, we adjusted for the oocyte maturation rate at retrieval in our current analysis to indicate the clinical application of ICSI with R-IVM. This study may be of great significance for patients with a high proportion of immature oocytes. Besides, two live births were achieved from 7 cycles that transfer embryos from R-IVM oocytes in women with DOR. Because of the small number of embryos for DOR patients, every additional embryo is of considerable potential clinical significance for them. Thus, these retrospective data suggest that ICSI with R-IVM is more successful, but larger retrospective and well-designed prospective studies are needed to confirm and reinforce the findings.

With respect to birth outcomes, all live births from R-IVM embryos were healthy and without malformations or complications. Studies have reported that the obstetric, perinatal, and neonatal outcomes and development of children conceived from IVM cycles seem similar to spontaneous conceptions or IVF treatment (12, 44, 45). Additionally, some studies have proved that an embryo from IVM oocytes could obtain a normal karyotype (46, 47). We should consider that data on IVM children are limited in our study, both in numbers and duration of follow-up. Further research about a more comprehensive appraisal of the health status of IVM children is still needed.

The strengths of this study include the relatively large sample size and the use of PSM and multivariable regression to minimize bias. However, some limitations should be noted. First, it was a retrospective cohort study conducted in a single-center, which may limit the external validity of our conclusions, as the heterogeneous practices between centers. The retrospective cohort study unlike randomized controlled trials (RCT), cannot control for unmeasured variables such as sperm concentration, sperm motility, and maternal comorbidities. Nevertheless, PSM presents a range of advantages over conventional regression models and some methodological similarities with RCTs. Second, data on pregnancy-related complications, congenital anomalies, and children’s development were not available. Third, small numbers of embryos from R-IVM oocytes were transferred; a future larger study is needed to compare the pregnancy outcomes between the two groups. Fourth, cycles with all mature oocytes were excluded, which comprised nearly one-third of the cycles at our center. Nevertheless, few studies have investigated differences in reproductive outcomes between only ICSI and ICSI with R-IVM. Therefore, our study results may provide useful insights for both clinicians and patients. Our study may provide immediate and long-term advantages by reducing the cancellation rate and providing extra blastocysts or transfers per attempt, and has the potential to give rise to pregnancies and live births, it may also provide a reference for future large-scale studies.

In conclusion, our study indicates that ICSI with R-IVM may increase pregnancy outcomes compared with only ICSI. It may also provide a reference for the safety of R-IVM. Adapting R-IVM as a routine may benefit the women undergoing ICSI. Cohort includes multiple centers worldwide and prospective design are needed.

## Data availability statement

The raw data supporting the conclusions of this article will be made available by the authors, without undue reservation.

## Ethics statement

This study was approved by the ethics committee of Tongji Hospital, Tongji Medical College, Huazhong University of Science and Technology. The patients/participants provided their written informed consent to participate in this study.

## Author contributions

D-YQ: Conceptualization, Methodology, Investigation, Formal analysis, Writing-original draft, Writing-review and editing; H-HJ: Investigation, Methodology, Formal analysis, Supervision, Writing-review and editing; Q-YY: Investigation, Methodology, Writing-review and editing; WY: Investigation, Writing-review and editing; X-QY: Investigation, Writing-review and editing; YW: Investigation, Data Curation; T-RD: Investigation, Writing-review and editing; Y-YD: Investigation, Methodology; X-LR: Investigation, Writing-review and editing; NG: Supervision, Investigation, Formal analysis, Methodology, and Writing-review and editing; Y-FL: Project administration, Conceptualization, Supervision, Writing-review and editing, Funding acquisition. All authors contributed to the article and approved the submitted version.
