# Supplementary material for: Rescue in vitro maturation may increase the pregnancy outcomes among women undergoing intracytoplasmic sperm injection
Source: Front Endocrinol (Lausanne). 2022 Dec 12;13:1047571. doi: 10.3389/fendo.2022.1047571 (PMC9790966; doi:10.3389/fendo.2022.1047571)
Supplement: Supplementary file 1 [file DataSheet_1.docx]

**Supplementary Materials**

**Rescue in vitro maturation may increase the pregnancy outcomes among women undergoing intracytoplasmic sperm injection**

Dan-Yu Qin, Hua-Hua Jiang, Qing-Yun Yao, Wen Yao, Xiao-Qiong Yuan, Yi Wang, Tao-Ran Deng, Yao-Yao Du, Xin-Ling Ren, Na Guo, Yu-Feng Li

***Supplementary Materials***

**Table of Contents**

**Table S1** Intermediate reproductive outcomes.

**Table S2** Pregnancy outcomes of fresh and frozen embryo transfer cycles.

**Table S3** Intermediate reproductive outcomes, stratified for in vivo matured and R-IVM oocytes separately in women undergoing ICSI with R-IVM.

**Table S4** Pregnancy and neonatal outcomes, stratified for in vivo matured and R-IVM oocytes separately in women undergoing ICSI with R-IVM.

**Table S5** Subgroup analysis of reproductive outcomes based on women with or without DOR.

**Table S6** Subgroup analysis of reproductive outcomes based on type of infertility.

**Table S7** Reproductive outcomes of women undergoing only ICSI versus women undergoing ICSI with R-IVM after excluding women with PCOS.

**Table S8** Reproductive outcomes of women undergoing only ICSI versus women undergoing ICSI with R-IVM after excluding cycles from couples diagnosed with male infertility.

**Figure legends**

**Figure S1** Flowchart of participants included in this study.

**Figure S2** Propensity score matching for women undergoing only ICSI and women undergoing ICSI with R-IVM. A, B The distribution of propensity scores. C, D The distribution of standard differences.

| **Table S1** Intermediate reproductive outcomes. | | | | | | |
| --- | --- | --- | --- | --- | --- | --- |
| Outcome | Before PSM | | | After PSM | | |
|  | Only ICSI | ICSI with  R-IVM |  | Only ICSI | ICSI with  R-IVM |  |
| Number | 2112 | 490 |  | 419 | 419 |  |
| MII oocytes | 10.8±6.1 | 10.0±5.6 |  | 9.0±5.5 | 10.6±5.6 |  |
| Oocyte maturation rate (%) | 75.2±13.7 | 78.4±16.3 |  | 66.1±16.4 | 80.7±14.5 |  |
| Normally fertilized embryos | 7.6±4.9 | 6.6±4.5 |  | 6.1±4.5 | 7.1±4.5 |  |
| Normal fertilization rate (%) | 68.9±22.3 | 64.2±21.7 |  | 66.9±26.8 | 65.6±21.1 |  |
| Cleaved embryos | 7.6±4.7 | 6.5±4.3 |  | 6.4±4.3 | 7.0±4.4 |  |
| Cleavage rate (%) | 97.6±8.0 | 96.4±12.5 |  | 97.8±7.9 | 96.7±12.0 |  |
| Good-quality embryos | 3.6±3.1 | 2.9±2.7 |  | 3.0±2.8 | 3.2±2.8 |  |
| Good-quality embryo rate (%) | 46.5±28.2 | 42.2±30.7 |  | 46.7±30.5 | 43.7±29.3 |  |
| Day-3 available embryos | 7.1±4.7 | 6.1±4.2 |  | 5.7±4.2 | 6.6±4.2 |  |
| Available embryo rate (%) | 49.2±19.1 | 46.7±21.3 |  | 42.4±20.9 | 49.2±20.6 |  |
| Embryos cultured past day-3 | 6.0±5.0 | 5.0±4.4 |  | 4.7±4.5 | 5.5±4.5 |  |
| Blastocysts | 4.6±3.9 | 3.9±3.4 |  | 4.0±3.4 | 4.2±3.5 |  |
| Blastocyst formation rate (%) | 59.5±30.8 | 55.8±33.9 |  | 59.2±32.6 | 58.0±32.3 |  |
| Available blastocysts | 3.3±3.0 | 2.7±2.7 |  | 2.8±2.6 | 2.9±2.7 |  |
| Available blastocyst rate (%) | 41.5±28.0 | 37.7±30.4 |  | 41.5±30.7 | 39.4±29.6 |  |
| Note: Data are presented as mean ± standard deviation. MII = metaphase 2; ICSI = intracytoplasmic sperm injection; R-IVM = rescue in vitro maturation. | | | | | | |

| **Table S2** Pregnancy outcomes of fresh and frozen embryo transfer cycles. | | | | | | |
| --- | --- | --- | --- | --- | --- | --- |
| Outcome | Before PSM | | | After PSM | | |
|  | Only ICSI | ICSI with  R-IVM | Adjusted OR (95% CI) | Only ICSI | ICSI with  R-IVM | OR (95% CI) |
| **Fresh ET (n)** | 1286 | 288 |  | 253 | 250 |  |
| No. of embryos  transferred | 1.1±0.4 | 1.2±0.4 |  | 1.2±0.4 | 1.1±0.4 |  |
| 1 | 1100(85.5) | 246(85.4) |  | 210(83.0) | 214(85.6) |  |
| 2 | 186(14.5) | 42(14.6) |  | 43(17.0) | 36(14.4) |  |
| Stage of embryos transferred |  |  |  |  |  |  |
| Cleavage embryo | 1153(89.7) | 269(93.4) |  | 233(92.1) | 231(92.4) |  |
| Blastocyst | 133(10.3) | 19(6.6) |  | 20(7.9) | 19(7.6) |  |
| Clinical pregnancy | 644(50.1) | 157(54.5) | **1.32 (1.00, 1.73)** ^a^ | 114(45.1) | 135(54.0) | **1.43 (1.01, 2.03)** |
| Miscarriage | 97(7.5) | 29(10.1) | 1.36 (0.86, 2.16) ^a^ | 16(6.3) | 24(9.6) | 1.57 (0.81, 3.04) |
| Live birth | 547(42.5) | 128(44.4) | 1.19 (0.91, 1.57) ^a^ | 98(38.7) | 111(44.4) | 1.26 (0.89, 1.80) |
| Singleton | 531(97.1) | 125(97.7) |  | 96(98.0) | 109(98.2) |  |
| Twin | 16(2.9) | 3(2.3) |  | 2(2.0) | 2(1.8) |  |
| **Frozen ET** |  |  |  |  |  |  |
| Total no. of FET with transfer | 1732 | 361 |  | 322 | 326 |  |
| No. of FET1 cycles | 1252 | 280 |  | 238 | 249 |  |
| No. of FET2 cycles | 355 | 66 |  | 65 | 63 |  |
| No. of FET3 cycles | 90 | 12 |  | 14 | 11 |  |
| No. of FET4 cycles | 26 | 2 |  | 4 | 2 |  |
| No. of FET5 cycles | 7 | 1 |  | 1 | 1 |  |
| No. of FET6 cycles | 2 | 0 |  | 0 | 0 |  |
| No. of embryos transferred | 1.3±0.4 | 1.3±0.4 |  | 1.3±0.4 | 1.3 ±0.4 |  |
| 1 | 1256(72.5) | 265(73.4) |  | 234(72.7) | 237(72.7) |  |
| 2 | 476(27.5) | 96(26.6) |  | 88(27.3) | 89(27.3) |  |
| Stage of embryos transferred |  |  |  |  |  |  |
| Cleavage embryo | 454(26.2) | 115(31.9) |  | 111(34.5) | 93(28.5) |  |
| Blastocyst | 1278(73.8) | 246(68.1) |  | 211(65.5) | 233(71.5) |  |
| Biochemical pregnancy | 113(6.5) | 26(7.2) |  | 17(5.3) | 24(7.4) |  |
| Clinical pregnancy | 930(53.7) | 190(52.6) |  | 158(49.1) | 178(54.6) |  |
| Miscarriage | 170(9.8) | 36(10.0) |  | 26(8.1) | 33(10.1) |  |
| Live birth | 739(42.7) | 150(41.6) |  | 132(41.0) | 141(43.3) |  |
| Singleton | 669(90.5) | 139(92.7) |  | 118(89.4) | 132(93.6) |  |
| Twin | 70(9.5) | 11(7.3) |  | 14(10.6) | 9(6.4) |  |
| Note: Data are presented as mean ± standard deviation or N (%). PSM = propensity score matching; ICSI = intracytoplasmic sperm injection; R-IVM = rescue in vitro maturation, OR= odds ratio.  ^a^ Adjusted for age, BMI, ovarian stimulation protocol, previous conventional IVF attempts, number of oocytes retrieved, oocyte maturation rate at retrieval, and stage of embryos transferred. | | | | | | |

| **Table S3** Intermediate reproductive outcomes, stratified for in vivo matured and R-IVM oocytes separately in women undergoing ICSI with R-IVM. | | | | | | |
| --- | --- | --- | --- | --- | --- | --- |
| Outcome | In vivo Matured oocytes | | R-IVM oocytes | | Combined R-IVM and in vivo matured oocytes | |
|  | n (%) | Mean ± SD  per patient | n (%) | Mean ± SD  per patient | n (%) | Mean ± SD  per patient |
| MII oocytes | 4065 | 8.3±5.6 | 822 | 1.7±1.3 | 4887 | 10.0±5.6* |
| Normally fertilized embryos | 2818 (69.3) | 5.8±4.3 | 397(48.3) | 0.8±1.0 | 3215(65.8)* | 6.6±4.5* |
| Cleaved embryos | 2751 (97.6) | 5.6±4.3 | 379(95.5) | 0.8±1.1 | 3130(97.4) | 6.4±4.4* |
| Good-quality embryos | 1240 (45.1) | 2.5±2.6 | 117(30.9) | 0.2±0.5 | 1357(43.4) | 2.8±2.7* |
| Day-3 available embryos | 2641 (65.0) | 5.4±4.1 | 437(53.2) | 0.7±0.9 | 3078(63.0) | 6.1±4.2* |
| Embryos cultured past day-3 | 2125 | 4.3±4.2 | 257 | 0.5±0.9 | 2382 | 4.9±4.4* |
| Blastocysts | 1368 (64.4) | 2.8±3.3 | 135(52.5) | 0.3±0.6 | 1503(63.1) | 3.1±3.4* |
| Available blastocysts | 963 (47.5) | 1.9±2.5 | 71(27.6) | 0.2±0.4 | 1034(43.4) | 2.1±2.6* |
| Note: Data are presented as mean ± standard deviation or N (%). MII = metaphase 2; ICSI = intracytoplasmic sperm injection; R-IVM = rescue in vitro maturation.  *Statistically significant, with *P* < 0.05. | | | | | | |

| **Table S4** Pregnancy and neonatal outcomes, stratified for in vivo matured and R-IVM oocytes separately in women undergoing ICSI with R-IVM. | | | |
| --- | --- | --- | --- |
| Outcome | In vivo Matured oocytes | R-IVM oocytes | Combined R-IVM and in vivo matured oocytes |
|  | n (%) | n (%) |  |
| **Pregnancy outcomes** |  |  |  |
| **Fresh ET (n)** | 269 | 14 | 288 |
| Clinical pregnancy | 149(55.4) | 6(42.9) | 157(54.5) |
| Miscarriage | 25(9.3) | 3(21.4) | 29(10.1) |
| Live birth | 124(46.1) | 3(21.4) | 128(44.4) |
| **The first ET (n)** | 408 | 42 | 462 |
| Clinical pregnancy | 221(54.2) | 18(42.9) | 245(53.0) |
| Miscarriage | 45(11.0) | 5(11.9) | 51(11.0) |
| Live birth | 176(43.1) | 13(31.0) | 194(42.0) |
| **Cumulative ET (n)** | 490 | 67 | 490 |
| Clinical pregnancy | 280(57.1) | 33(49.3) | 322(65.7)* |
| Miscarriage | 37(7.6) | 6(9.0) | 44(9.0) |
| Live birth | 243(49.6) | 27(40.3) | 278(56.7)* |
| Singleton | 233(95.9) | 24(88.9) | 264(95.0) |
| Twin | 10(4.1) | 3(11.1) | 14(5.0) |
| **Neonatal outcomes** |  |  |  |
| Gestational age (weeks) | 38.1±2.0 | 37.1±2.2 | 38.0±2.0 |
| Birth weight (g) | 3209.3±567.3 | 3178.9±654.3 | 3199.8±571.1 |
| Preterm birth < 37 wk | 36(14.8) | 7(25.9) | 45(16.2) |
| Macrosomia > 4,000 g | 13(5.3) | 2(7.4) | 15(5.4) |
| Low birth weight < 2,500 g | 23(9.5) | 3(11.1) | 26(9.4) |
| **Singleton** |  |  |  |
| Gestational age (weeks) | 38.2±1.8 | 37.4±2.2 | 38.1±1.9 |
| Birth weight (g) | 3249.2±534.9 | 3280.4±614.1 | 3244.9±539.3 |
| Preterm birth < 37 wk | 28(12.0) | 4(16.7) | 34(12.9) |
| Macrosomia > 4,000 g | 13(5.6) | 2(8.3) | 15(5.7) |
| Low birth weight < 2,500 g | 15(6.4) | 1(4.2) | 16(6.1) |
| Note: Data are presented as mean ± standard deviation or N (%). ICSI = intracytoplasmic sperm injection; R-IVM = rescue in vitro maturation.  *Statistically significant, with *P* < 0.05. | | | |

| **Table S5** Subgroup analysis of reproductive outcomes based on women with or without DOR. | | | | | | |
| --- | --- | --- | --- | --- | --- | --- |
| Outcome | With DOR | | | Without DOR | | |
|  | Only ICSI | ICSI with  R-IVM | Percent change/OR (95%CI) | Only ICSI | ICSI with  R-IVM | Percent change/OR (95%CI) |
| Number | 63 | 58 |  | 356 | 361 |  |
| MII oocytes | 4.0±3.0 | 5.3±2.8 | **32.4(12.1, 56.5)** | 9.9±5.4 | 11.5±5.5 | **16.3(11.2, 21.7)** |
| Oocyte maturation rate(%) | 58.3±17.6 | 81.5±17.1 | **109.8(53.3, 187.1)** | 67.5±15.8 | 80.6±14.1 | **80.7(65.2, 97.6)** |
| Normally fertilized embryos | 2.6±2.6 | 3.1±2.3 | 17.1(–5.2, 44.7) | 6.8±4.4 | 7.7±4.5 | **14.5 (8.4, 20.9)** |
| Normal fertilization rate(%) | 61.1±37.9 | 57.8±26.2 | –27.8(–49.0, 2.1) | 67.9±24.2 | 66.9±19.9 | –3.5(–12.4, 6.3) |
| Cleaved embryos | 3.3±2.4 | 3.1±2.3 | –7.1(–25.0, 15.2) | 6.8±4.3 | 7.6±4.3 | **11.8 (5.8, 18.1)** |
| Cleavage rate(%) | 99.0±3.9 | 93.7±20.9 | –65.2(–90.8, 30.7) | 97.6±8.3 | 97.1±9.9 | –14.1(–40.2, 23.5) |
| Good-quality embryos | 1.9±1.5 | 1.4±1.5 | –26.9(–46.3, 0.4) | 3.2±2.9 | 3.4±2.8 | 8.0 (–0.5, 17.2) |
| Good-quality embryo rate(%) | 59.3±36.2 | 40.2±38.9 | –41.9(–62.3, 10.3) | 45.0±29.2 | 44.2±27.6 | –6.6(–16.4, 4.3) |
| Day-3 available embryos | 2.5±2.4 | 2.8±2.3 | 12.1(–10.0, 39.6) | 6.3±4.2 | 7.2±4.2 | **14.0(7.7, 20.6)** |
| Available embryo rate (%) | 35.3±24.4 | 41.8±24.4 | 8.6(–18.3, 44.3) | 43.6±20.0 | 50.4±19.6 | **29.3(19.7, 39.7)** |
| Embryos cultured past day-3 | 1.7±2.4 | 1.6±2.3 | –10.1(–33.4, 21.3) | 5.2±4.5 | 6.0±4.4 | **17.1(10.0, 24.7)** |
| Blastocysts | 2.2±1.7 | 1.8±2.0 | –17.5(–43.8, 21.1) | 4.2±3.5 | 4.4±3.5 | 4.7(–3.1, 13.2) |
| Blastocyst formation rate(%) | 58.6±35.9 | 47.3±40.6 | –6.6(–49.5, 72.6) | 59.3±32.4 | 58.9±31.3 | –5.7(–17.3, 7.5) |
| Available blastocysts | 1.7±1.6 | 1.1±1.7 | –33.1(–57.9, 6.2) | 2.9±2.7 | 3.0±2.7 | 4.3(–4.9, 14.5) |
| Available blastocyst rate(%) | 46.6±37.0 | 26.9±36.1 | –33.6(–64.0, 21.9) | 41.0±30.2 | 40.5±28.7 | –4.6(–15.6, 8.5) |
| **Pregnancy outcomes** |  |  |  |  |  |  |
| **Fresh ET (n)** | 26 | 16 |  | 227 | 234 |  |
| Clinical pregnancy | 9(34.6) | 4(25.0) | 0.63 (0.16, 2.53) | 105(46.3) | 131(56.0) | 1.48 (1.02, 2.13) |
| Miscarriage | 2(7.7) | 0 |  | 14(6.2) | 24(10.3) | 1.74 (0.88, 3.45) |
| Live birth | 7(26.9) | 4(25.0) | 0.91 (0.22, 3.76) | 91(40.1) | 107(45.7) | 1.26 (0.87, 1.82) |
| **The first ET (n)** | 63 | 58 |  | 340 | 349 |  |
| Biochemical pregnancy | 1(2.1) | 2(4.0) | 1.92 (0.17, 21.9) | 8(2.4) | 14(4.0) | 1.73 (0.72, 4.19) |
| Clinical pregnancy | 13(27.7) | 14(28.0) | 1.02 (0.42, 2.47) | 154(45.3) | 201 (57.6) | **1.64 (1.21, 2.22)** |
| Miscarriage | 3(6.4) | 4(8.0) | 1.28 (0.27, 6.03) | 19(5.6) | 40(11.5) | **2.19 (1.24, 3.86)** |
| Live birth | 10(21.3) | 10(20.0) | 0.93 (0.35, 2.47) | 135(39.7) | 161(46.1) | 1.30 (0.96, 1.76) |
| **Cumulative outcome (n)** | 63 | 58 |  | 356 | 361 |  |
| Biochemical pregnancy | 3(4.8) | 2(3.4) | 0.71 (0.12, 4.43) | 13(3.7) | 22(6.1) | 1.71 (0.85, 3.46) |
| Clinical pregnancy | 19(30.2) | 18(31.0) | 1.04 (0.48, 2.26) | 237(66.6) | 272(75.3) | **1.54 (1.11, 2.12)** |
| Miscarriage | 3(4.8) | 4(6.9) | 1.48 (0.32, 6.92) | 23(6.5) | 34(9.4) | 1.51 (0.87, 2.61) |
| Cumulative live birth | 16(25.4) | 14 (24.1) | 0.94 (0.41, 2.14) | 214(60.1) | 238(65.9) | 1.28 (0.96, 1.74) |
| Note: Data are presented as mean ± standard deviation. DOR = diminished ovarian reserve; MII = metaphase 2; ICSI = intracytoplasmic sperm injection; R-IVM = rescue in vitro maturation; OR= odds ratio. | | | | | | |

| **Table S6** Subgroup analysis of reproductive outcomes based on type of infertility. | | | | | | |
| --- | --- | --- | --- | --- | --- | --- |
| Outcome | Primary | | | Secondary | | |
|  | Only ICSI | ICSI with  R-IVM | Percent change/OR (95%CI) | Only ICSI | ICSI with  R-IVM | Percent change/OR (95%CI) |
| Number | 317 | 316 |  | 102 | 103 |  |
| MII oocytes | 9.2±5.6 | 11.1±5.7 | **21.1 (15.3, 27.2)** | 8.4±5.0 | 9.1±5.2 | 8.7 (–1.0, 19.2) |
| Oocyte maturation rate(%) | 65.6±16.9 | 80.6±14.4 | **86.0 (68.7, 104.9)** | 67.7±14.6 | 80.9±14.9 | **71.9 (42.6, 107.2)** |
| Normally fertilized embryos | 6.3±4.6 | 7.4±4.7 | **16.9 (10.1, 24.1)** | 5.5±4.0 | 6.1±3.9 | 10.9 (–1.0, 24.3) |
| Normal fertilization rate(%) | 68.0±26.3 | 65.4±20.8 | –8.9 (–18.0, 1.2) | 63.5±28.1 | 66.5±22.1 | –6.3 (–12.6, 29.2) |
| Cleaved embryos | 6.5±4.4 | 7.3±4.5 | **12.4 (5.8, 19.4)** | 5.9±3.8 | 6.0±4.0 | 2.0 (–10.1, 14.5) |
| Cleavage rate(%) | 97.7±6.8 | 97.1±9.3 | –12.9 (–40.4, 27.2) | 98.1±10.8 | 95.5±17.8 | –48.3 (–79.1, 27.6) |
| Good-quality embryos | 3.0±2.8 | 3.3±2.8 | **10.9 (1.4, 21.2)** | 3.0±2.6 | 2.6±2.7 | –13.4 (–26.8, 2.5) |
| Good-quality embryo rate(%) | 45.3±30.3 | 44.4±28.7 | –3.0 (–14.1, 9.5) | 51.4±30.6 | 41.4±31.3 | –29.1 (–43.8, 10.7) |
| Day-3 available embryos | 5.9±4.3 | 6.9±4.3 | **16.8 (9.8, 24.3)** | 5.2±3.9 | 5.6±3.9 | 7.8 (–4.2, 21.3) |
| Available embryo rate (%) | 42.5±20.6 | 49.7±20.4 | **28.0 (17.6, 39.3)** | 42.1±21.9 | 47.8±21.0 | **27.0 (8.2, 49.2)** |
| Embryos cultured past day-3 | 4.9±4.6 | 5.8±4.6 | **17.7 (9.9, 26.1)** | 4.2±4.1 | 4.5±4.0 | 7.0 (–6.5, 22.4) |
| Blastocysts | 4.1±3.5 | 4.4±3.4 | 6.7 (–2.0, 16.2) | 3.7±3.1 | 3.5±3.4 | –6.9 (–21.3, 10.1) |
| Blastocyst formation rate(%) | 58.7±32.8 | 59.7±31.2 | –3.5 (–16.4, 11.4) | 60.9±32.4 | 52.4±35.1 | –13.3 (–34.7, 15.1) |
| Available blastocysts | 2.8±2.7 | 3.1±2.7 | 7.8 (–2.6, 19.5) | 2.7±2.5 | 2.3±2.7 | –15.7 (–31.1, 3.2) |
| Available blastocyst rate(%) | 40.8±30.9 | 41.8±28.8 | –0.4 (–13.2, 14.3) | 43.8±30.0 | 31.8±30.7 | –23.7 (–41.9, 0.2) |
| **Pregnancy outcomes** |  |  |  |  |  |  |
| **Fresh ET (n)** | 195 | 195 |  | 58 | 55 |  |
| Clinical pregnancy | 85(43.6) | 110(56.4) | **1.68(1.12, 2.50)** | 29(50.0) | 25(45.5) | 0.83(0.40, 1.75) |
| Miscarriage | 11(5.6) | 16(8.2) | 1.50(0.68, 3.31) | 5(8.6) | 8(14.5) | 1.80(0.55, 5.90) |
| Live birth | 74(37.9) | 94(48.2) | **1.52(1.02, 2.28)** | 24(41.4) | 17(30.9) | 0.63(0.29, 1.38) |
| **The first ET (n)** | 294 | 304 |  | 93 | 95 |  |
| Biochemical pregnancy | 8(2.7) | 13(4.3) | 1.60(0.65, 3.91) | 1(1.1) | 3(3.2) | 3.00(0.31, 29.4) |
| Clinical pregnancy | 127(43.2) | 172(56.6) | **1.71 (1.24, 2.37)** | 40(43.0) | 43(45.3) | 1.10(0.62, 1.95) |
| Miscarriage | 15(5.1) | 28(9.2) | 1.89(0.99, 3.61) | 7(7.5) | 16(16.8) | 2.49(0.97, 6.37) |
| Live birth | 112(38.1) | 144(47.4) | **1.46(1.06, 2.03)** | 33(35.5) | 27(28.4) | 0.72(0.39, 1.34) |
| **Cumulative outcome (n)** | 317 | 316 |  | 102 | 103 |  |
| Biochemical pregnancy | 12 (3.8) | 19(6.0) | 1.63(0.78, 3.41) | 4(3.9) | 5(4.9) | 1.25(0.33, 4.79) |
| Clinical pregnancy | 198(62.5) | 231(73.1) | **1.63(1.17, 2.29)** | 58(56.9) | 59(57.3) | 1.02(0.59, 1.77) |
| Miscarriage | 19(6.0) | 22 (7.0) | 1.17 (0.62, 2.21) | 7(6.9) | 16(15.5) | 2.50(0.98, 6.36) |
| Cumulative live birth | 179(56.5) | 209(66.1) | **1.51(1.09, 2.08)** | 51(50.0) | 43(41.7) | 0.72(0.41, 1.24) |
| Note: Data are presented as mean ± standard deviation. MII = metaphase 2; ICSI = intracytoplasmic sperm injection; R-IVM = rescue in vitro maturation; OR= odds ratio. | | | | | | |

| **Table S7** Reproductive outcomes of women undergoing only ICSI versus women undergoing ICSI with R-IVM after excluding women with PCOS. | | | |
| --- | --- | --- | --- |
| Outcome | Only ICSI | ICSI with R-IVM | Percent change/OR (95%CI) |
| **Intermediate reproductive outcomes** |  |  |  |
| Number | 387 | 384 |  |
| MII oocytes | 8.7±5.3 | 10.3±5.5 | **18.3 (13.0, 23.8)** |
| Oocyte maturation rate (%) | 65.8±16.5 | 80.8±14.5 | **88.0 (71.7, 105.9)** |
| Normally fertilized embryos | 5.9±4.4 | 6.9±4.4 | **16.1 (9.8, 22.8)** |
| Normal fertilization rate (%) | 66.3±27.3 | 65.7±20.7 | –4.0 (–13.0, 5.9) |
| Cleaved embryos | 6.2±4.2 | 6.8±4.2 | **9.7 (3.7, 16.1)** |
| Cleavage rate (%) | 97.7±8.2 | 96.6±12.3 | –20.4 (–44.8, 14.6) |
| Good-quality embryos | 2.9±2.7 | 3.1±2.8 | 5.9 (–2.6, 15.1) |
| Good-quality embryo rate (%) | 46.9±30.9 | 43.3±29.9 | –7.8 (–17.7, 3.3) |
| Day-3 available embryos | 5.6±4.1 | 6.4±4.1 | **15.9 (9.4, 22.8)** |
| Available embryo rate (%) | 41.9±21.1 | 49.3±20.3 | **32.2 (22.1, 43.0)** |
| Embryos cultured past day-3 | 4.6±4.3 | 5.3±4.4 | **16.5 (9.1, 24.3)** |
| Blastocysts | 3.9±3.3 | 4.0±3.4 | 3.7 (–4.3, 12.5) |
| Blastocyst formation rate (%) | 59.1±32.5 | 57.4±32.8 | –4.4 (–16.5, 9.6) |
| Available blastocysts | 2.7±2.5 | 2.9±2.7 | 5.2 (–4.5, 15.9) |
| Available blastocyst rate (%) | 41.2±30.8 | 39.8±29.8 | –0.3 (–12.5, 13.7) |
| **Pregnancy outcomes** |  |  |  |
| **Fresh ET (n)** | 232 | 230 |  |
| Clinical pregnancy | 106(45.7) | 122 (53.0) | 1.34 (0.93, 1.94) |
| Miscarriage | 14(6.0) | 23(10.0) | 1.73 (0.87, 3.45) |
| Live birth | 92(39.7) | 99 (43.0) | 1.15 (0.79, 1.67) |
| **The first ET (n)** | 355 | 365 |  |
| Biochemical pregnancy | 9(2.5) | 14(3.8) | 1.53 (0.66, 3.59) |
| Clinical pregnancy | 155(43.7) | 194(53.2) | **1.46 (1.09, 1.96)** |
| Miscarriage | 20(5.6) | 41(11.2) | **2.12 (1.22, 3.70)** |
| Live birth | 135(38.0) | 153(41.9) | 1.18 (0.87, 1.59) |
| **Cumulative outcome (n)** | 387 | 384 |  |
| Biochemical pregnancy | 16(4.1) | 22(5.7) | 1.41 (0.73, 2.73) |
| Clinical pregnancy | 234(60.5) | 264(68.8) | **1.44 (1.07, 1.94)** |
| Miscarriage | 22(5.7) | 34(8.9) | 1.61 (0.92, 2.81) |
| Cumulative live birth | 212(54.8) | 230(59.9) | 1.23 (0.93, 1.64) |
| Note: Data are presented as mean ± standard deviation or N (%). MII = metaphase 2; ICSI = intracytoplasmic sperm injection; R-IVM = rescue in vitro maturation; OR= odds ratio. | | | |

| **Table S8** Reproductive outcomes of women undergoing only ICSI versus women undergoing ICSI with R-IVM after excluding cycles from couples diagnosed with male infertility. | | | |
| --- | --- | --- | --- |
| Outcome | Only ICSI | ICSI with R-IVM | Percent change/OR (95%CI) |
| **Intermediate reproductive outcomes** |  |  |  |
| Number | 240 | 239 |  |
| MII oocytes | 8.1±5.6 | 9.8±5.8 | **20.7 (13.7, 28.2)** |
| Oocyte maturation rate (%) | 64.8±16.8 | 79.5±15.2 | **67.3 (48.9, 87.9)** |
| Normally fertilized embryos | 5.6±4.6 | 6.6±4.7 | **17.0 (8.8, 25.9)** |
| Normal fertilization rate (%) | 66.7±29.1 | 65.0±22.6 | –6.8 (–18.1, 6.0) |
| Cleaved embryos | 6.0±4.4 | 6.5±4.6 | **8.8 (1.1, 17.1)** |
| Cleavage rate (%) | 98.4±6.2 | 95.9±14.9 | –39.8 (–63.8, 0.3) |
| Good-quality embryos | 2.9±2.8 | 3.0±2.7 | 2.6 (–7.8, 14.3) |
| Good-quality embryo rate (%) | 48.8±31.6 | 44.1±30.2 | –13.3 (–25.2, 0.5) |
| Day-3 available embryos | 5.3±4.4 | 6.1±4.4 | **14.8 (6.4, 23.7)** |
| Available embryo rate (%) | 41.9±22.1 | 47.7±21.4 | **19.7 (8.0, 32.7)** |
| Embryos cultured past day-3 | 4.3±4.6 | 4.9±4.6 | **14.1 (4.7, 24.3)** |
| Blastocysts | 3.9±3.7 | 3.9±3.6 | 0.6 (–9.6, 11.9) |
| Blastocyst formation rate (%) | 57.4±33.9 | 54.9±33.0 | –11.0 (–25.5, 6.5) |
| Available blastocysts | 2.8±2.8 | 2.6±2.7 | –6.3 (–17.7, 6.6) |
| Available blastocyst rate (%) | 41.3±31.2 | 35.5±28.9 | –18.2 (–31.1, 2.7) |
| **Pregnancy outcomes** |  |  |  |
| **Fresh ET (n)** | 140 | 122 |  |
| Clinical pregnancy | 56(40.0) | 59(48.4) | 1.41 (0.86, 2.29) |
| Miscarriage | 10(7.1) | 11(9.0) | 1.29 (0.53, 3.15) |
| Live birth | 46(32.9) | 48(39.3) | 1.33 (0.80, 2.20) |
| **The first ET (n)** | 217 | 223 |  |
| Biochemical pregnancy | 3(1.4) | 9(4.0) | 3.00 (0.80, 11.2) |
| Clinical pregnancy | 80(36.9) | 107(48.0) | **1.58 (1.08, 2.31)** |
| Miscarriage | 12(5.5) | 23(10.3) | 1.97 (0.95, 4.06) |
| Live birth | 68(31.3) | 84(37.7) | 1.32 (0.89, 1.97) |
| **Cumulative outcome (n)** | 240 | 239 |  |
| Biochemical pregnancy | 8(3.3) | 13(5.4) | 1.67 (0.68, 4.10) |
| Clinical pregnancy | 130(54.2) | 148(61.9) | 1.38 (0.96, 1.98) |
| Miscarriage | 18(7.5) | 24(10.0) | 1.38 (0.73, 2.61) |
| Cumulative live birth | 112(46.7) | 124 (51.9) | 1.23 (0.86, 1.76) |
| Note: Data are presented as mean ± standard deviation or N (%). MII = metaphase 2; ICSI = intracytoplasmic sperm injection; R-IVM = rescue in vitro maturation; OR= odds ratio. | | | |


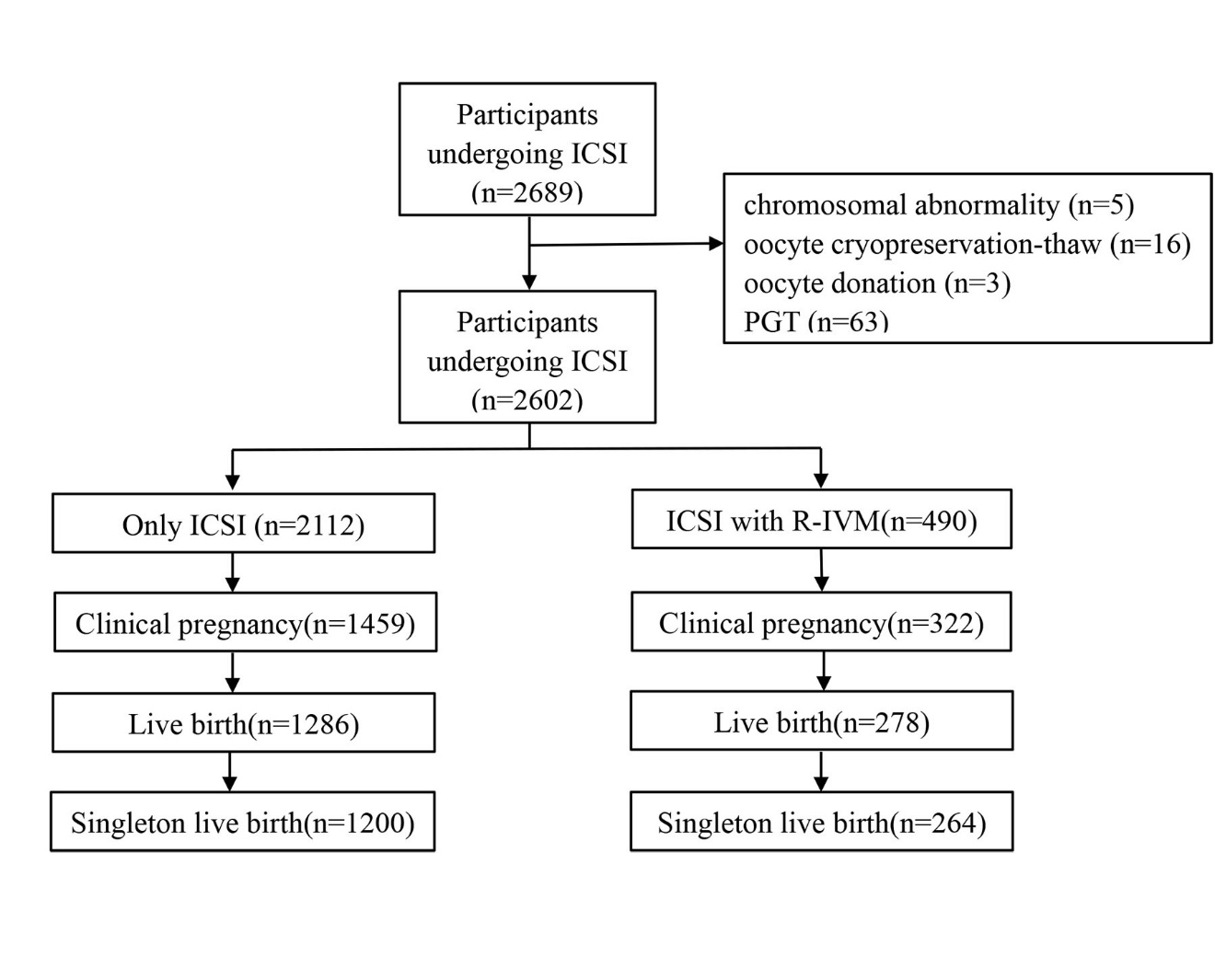


**Figure S1** Flowchart of participants included in this study.


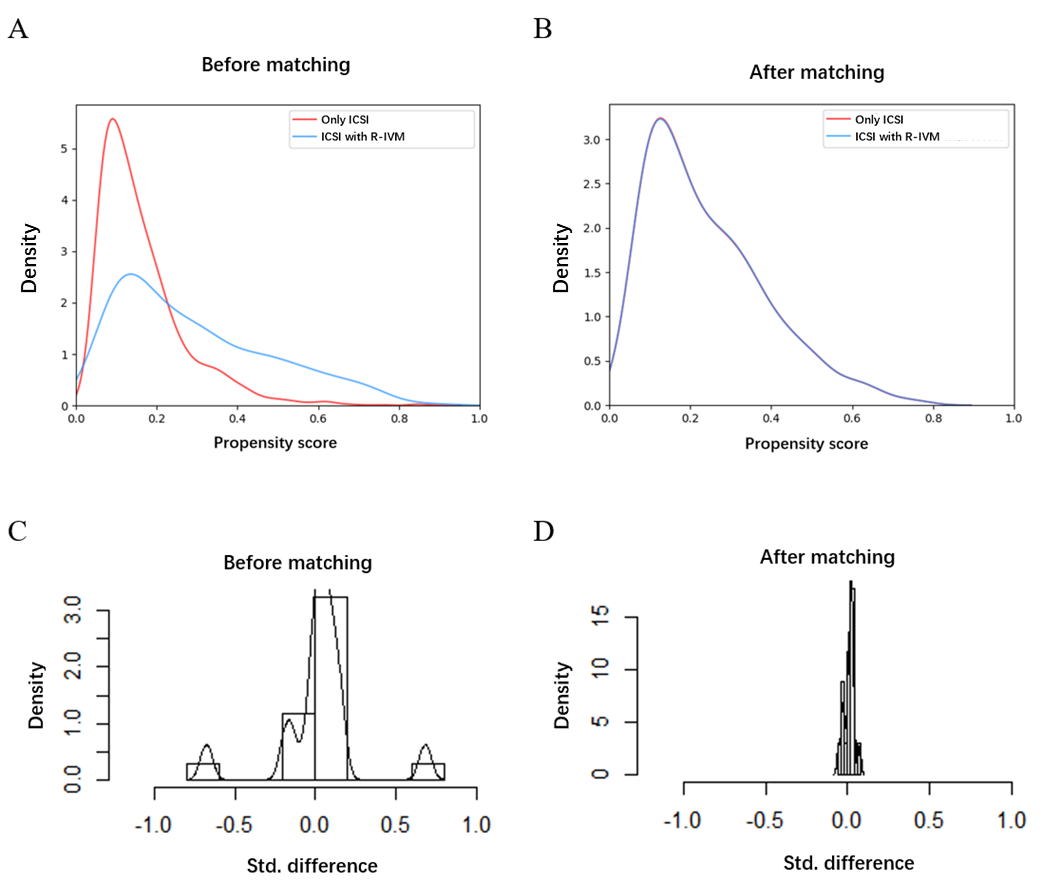


**Figure S2** Propensity score matching for women undergoing only ICSI and women undergoing ICSI with R-IVM. A, B The distribution of propensity scores. C, D The distribution of standard differences.
